# Racial and Demographic Disparities in Susceptibility to Health Misinformation on Social Media: National Survey-Based Analysis

**DOI:** 10.2196/55086

**Published:** 2024-11-06

**Authors:** Ranganathan Chandrasekaran, Muhammed Sadiq T, Evangelos Moustakas

**Affiliations:** 1 University of Illinois at Chicago Chicago, IL United States; 2 Indian Institute of Technology Madras Chennai India; 3 Canadian University Dubai Dubai United Arab Emirates

**Keywords:** health misinformation, digital divide, racial disparities, social media, national survey-based analysis, health information, interventions

## Abstract

**Background:**

Social media platforms have transformed the dissemination of health information, allowing for rapid and widespread sharing of content. However, alongside valuable medical knowledge, these platforms have also become channels for the spread of health misinformation, including false claims and misleading advice, which can lead to significant public health risks. Susceptibility to health misinformation varies and is influenced by individuals’ cultural, social, and personal backgrounds, further complicating efforts to combat its spread.

**Objective:**

This study aimed to examine the extent to which individuals report encountering health-related misinformation on social media and to assess how racial, ethnic, and sociodemographic factors influence susceptibility to such misinformation.

**Methods:**

Data from the Health Information National Trends Survey (HINTS; Cycle 6), conducted by the National Cancer Institute with 5041 US adults between March and November 2022, was used to explore associations between racial and sociodemographic factors (age, gender, race/ethnicity, annual household income, marital status, and location) and susceptibility variables, including encounters with misleading health information on social media, difficulty in assessing information truthfulness, discussions with health providers, and making health decisions based on such information.

**Results:**

Over 35.61% (1740/4959) of respondents reported encountering “a lot” of misleading health information on social media, with an additional 45% (2256/4959) reporting seeing “some” amount of health misinformation. Racial disparities were evident in comparison with Whites, with non-Hispanic Black (odds ratio [OR] 0.45, 95% CI 0.33-0.6, *P*<.01) and Hispanic (OR 0.54, 95% CI 0.41-0.71, *P*<.01) individuals reporting lower odds of finding deceptive information, while Hispanic (OR 1.68, 95% CI 1.48-1.98, *P*<.05) and non-Hispanic Asian (OR 1.96, 95% CI 1.21-3.18, *P*<.01) individuals exhibited higher odds in having difficulties in assessing the veracity of health information found on social media. Hispanic and Asian individuals were more likely to discuss with providers and make health decisions based on social media information. Older adults aged ≥75 years exhibited challenges in assessing health information on social media (OR 0.63, 95% CI 0.43-0.93, *P*<.01), while younger adults (18-34) showed increased vulnerability to health misinformation. In addition, income levels were linked to higher exposure to health misinformation on social media: individuals with annual household incomes between US $50,000 and US $75,000 (OR 1.74, 95% CI 1.14-2.68, *P*<.01), and greater than US $75,000 (OR 1.78, 95% CI 1.20-2.66, *P*<.01) exhibited greater odds, revealing complexities in decision-making and information access.

**Conclusions:**

This study highlights the pervasive presence of health misinformation on social media, revealing vulnerabilities across racial, age, and income groups, underscoring the need for tailored interventions.

## Introduction

### Background

In today’s digitally connected world, social media has emerged as a fertile platform for the rapid dissemination of health-related content. Platforms such as Facebook [1], Instagram [2], YouTube [3], and Twitter [4] offer users the ability to share personal health experiences, disseminate medical news, and engage in online communities centered on health and wellness. This quick and widespread distribution of health information has the potential to elevate public health discourse, fostering awareness and encouraging dialogue, as was witnessed during the COVID-19 pandemic [5]. However, alongside the abundance of genuine and reliable health information, there is also a growing concern regarding the proliferation of misinformation on social media platforms [6,7]. Health-related misinformation, including rumors, pseudoscience, and personal anecdotes, can rapidly spread, often influencing unsuspecting users [8]. This misinformation can range from misleading claims about miracle cures to false opinions about vaccines, leading individuals to potentially harmful or dangerous practices [9,10].

Susceptibility to misinformation refers to an individual’s tendency or vulnerability to believe, accept, or be influenced by false or misleading information, often despite evidence to the contrary. This susceptibility can stem from various factors, including cognitive biases, lack of critical thinking skills, emotional responses, social influence, and the persuasive techniques used by purveyors of misinformation [11]. Extant research suggests that certain segments of the population, such as individuals with lower health literacy, higher educational attainment, or those seeking alternative therapies [12], may be particularly susceptible to health-related misinformation on social media [13-16]. Given the far-reaching impact of social media on individuals’ perceptions and behaviors, understanding the vulnerabilities that make certain individuals more prone to accepting and sharing health misinformation is crucial. It becomes important to identify the most vulnerable groups and target specific interventions to combat health misinformation [17].

Current studies on health misinformation on social media have documented the diffusion patterns [18,19], nature, and type of health misinformation and have suggested negative outcomes from such misinformation spread [17,20,21], including distorted interpretations of medical evidence, negative impacts on mental well-being, misallocation of health care resources, and a rise in vaccine hesitancy [22]. Furthermore, studies have also proposed advanced machine learning and artificial intelligence–based models that could detect health misinformation on social media [23-25]. However, limited research has delved into understanding the characteristics of individuals who are prone to accepting and using health misinformation [26]. Our study addresses this gap by documenting the nature and type of individuals who are more susceptible to health misinformation on social media.

Our research aims to explore 2 key questions. First, we seek to understand what kinds of individuals are more prone to accepting and believing health misinformation on social media. We specifically explore whether there are racial differences and other disparities in the susceptibility of individuals to health fake news content on social media. Recognizing unequal vulnerabilities and disparities in health information exposure and decision-making is essential for ensuring equitable access to accurate health information and promoting health equity.

Second, we intend to investigate how individuals react to health misinformation encountered on social media. We assess if they include such information in their discussions with providers or how such information affects their decisions pertaining to their health. Understanding the cognitive and emotional responses individuals have when presented with false or misleading health information is essential to comprehending the potential impact on their decision-making processes, health behaviors, and overall well-being.

### Theoretical Underpinnings

Selective and superficial information processing represent 2 prominent theoretical frames within the realm of cognitive psychology, offering insights into how individuals perceive, evaluate, and act upon information encountered in various contexts. These 2 theoretical frames provide valuable perspectives for understanding susceptibility to misinformation, particularly in the context of health information dissemination on social media platforms.

Selective information processing posits that individuals tend to selectively attend to and interpret information in a manner that is congruent with their existing beliefs, attitudes, and motivations [27,28]. This selective attention and interpretation of information allow individuals to maintain consistency within their cognitive frameworks and protect their preexisting beliefs from cognitive dissonance [29,30]. Selective information processing is driven by various factors, including cognitive biases, emotional responses, social influences, and motivational goals [31]. Individuals engaging in selective information processing may exhibit confirmation bias, wherein they prioritize information that confirms their preconceptions while discounting or ignoring contradictory evidence [32,33].

Contrary to selective processing, superficial information processing involves the use of cognitive shortcuts or heuristics to evaluate information, often leading to quick and intuitive judgments rather than using thorough analysis [34,35]. This mode of information processing is characterized by minimal cognitive effort and reliance on mental shortcuts, such as heuristics, to make judgments or decisions [36]. Superficial processing is influenced by factors such as cognitive load, time constraints, and cognitive fluency [37,38]. Individuals engaging in superficial information processing may be susceptible to misinformation due to their tendency to overlook nuances, inconsistencies, or inaccuracies in the information presented [11].

The concepts of selective and superficial information processing guided us to choose specific variables for assessing individuals’ susceptibility to health misinformation on social media, as explained in the next section.

## Methods

This study relies on data sourced from the Health Information National Trends Survey (HINTS)— a comprehensive national survey conducted by the National Cancer Institute. HINTS is designed to provide nationally representative data, offering insights into the health-related needs, social media access and use, perceptions, and knowledge of adults aged ≥18 years in the United States [39]. HINTS 6, the latest iteration of this survey, includes a comprehensive set of questions pertaining to social media use, health misinformation on social media, and demographic factors. HINTS 6 follows a biennial data collection schedule, using both paper and web-based survey modes. The data collection for HINTS 6 commenced on March 7, 2022, and concluded on November 8, 2022. The final data set of HINTS 6 encompasses responses from 6252 participants, rendering it a robust resource for our research endeavors. Control totals in HINTS 6 underwent adjustments by drawing insights from the 2021 American Community Survey (ACS) carried out by the US Census Bureau. These adjustments factored in a range of variables, including age, gender, educational attainment, marital status, as well as racial and ethnic backgrounds. This was instrumental in harmonizing the demographic attributes within the HINTS 6 data set to closely mirror the population characteristics identified by the Census Bureau so that population-level estimates can be computed.

In the HINTS survey, weighted frequencies are calculated by applying survey weights to each respondent’s data. These weights adjust for differences in the probability of selection and nonresponse rates across demographic groups, ensuring that the survey results are representative of the target population. The weights are derived from statistical procedures that consider sampling design, survey nonresponse, and demographic distributions from external sources such as census data. Applying these weights helps provide accurate estimates of population parameters in the survey results. Additional information is available in the HINTS methodology report [40].

### Variables

We used a set of 4 questions from the HINTS 6 survey to gauge individuals’ susceptibility to health misinformation on social media platforms. First, respondents were asked to assess the credibility of health information encountered on social media by rating the question, “How much of the health information that you see on social media do you think is false or misleading?” with response options ranging from 1 (none) to 4 (a lot). Second, respondents were asked to rate the level of difficulty they encountered in discerning the truthfulness of health information on social media using the question, “I find it hard to tell whether health information on social media is true or false,” with response options ranging from “strongly disagree” to “strongly agree.” Third, we sought to understand participants’ agreement or disagreement with statements related to their use of health information on social media through 2 questions: one inquiring whether they used social media data for making personal health-related decisions (“I use information from social media to make decisions about my health”), another regarding the use of social media information in their discussions with health care providers (“I use information from social media in discussions with my healthcare provider”). Respondents indicated their agreement with these statements using a scale ranging from “strongly disagree” to “strongly agree.”

The 4 facets used in this study–perception of the frequency of false health information on social media, difficulty in discerning the truthfulness of health information, use of social media data for personal health decisions, and use of social media information in discussions with health care providers–can be linked to the theoretical concepts of selective and superficial information processing.

Perception of false health information on social media and use of social media data for personal health decisions reflect individuals’ engagement in selective information processing. Individuals who perceive a significant portion of health information on social media as false or misleading and rely heavily on social media data for personal health decisions are likely to selectively attend to information that confirms their preexisting beliefs or biases. Similarly, the use of social media information in discussions with health care providers may also be driven by selective processing, wherein individuals prioritize information that aligns with their beliefs or preferences while disregarding contradictory evidence.

On the other hand, difficulty in discerning the truthfulness of health information may indicate engagement in superficial information processing. Individuals experiencing great difficulty in evaluating the truthfulness of health information on social media may resort to cognitive shortcuts or heuristics, leading to superficial judgments that overlook the accuracy or validity of the information presented.

To assess variations in susceptibility to health misinformation on social media due to sociodemographic disparities, we chose a set of variables for our analysis. These factors encompassed age, which was categorized into 5 groups: 18-34, 35-49, 50-64, 65-74, and ≥75 years. We also considered birth gender (male or female) and race/ethnicity, which we classified as non-Hispanic White, non-Hispanic Black/African American, Hispanic, non-Hispanic Asian, or other. Household income was divided into 5 categories: <US $20,000, US $20,000-<US $35,000, US $35,000-<US $50,000, US $50,000-<US $75,000, and ≥US $75,000. Education levels were grouped as less than high school, high school graduate, some college, or college graduate or more. Marital status was categorized as married or otherwise. In addition, we classified the nature of residence using the National Center for Health Statistics (NCHS) 2013 Rural-Urban Commuting Area (RUCA) system, which assigns RUCA codes to specific census tracts based on factors such as population density, urbanization, and commuting patterns. These RUCA codes encompassed various residential categories, including large metro, large fringe metro, medium metro, small metro, nonmetropolitan micropolitan, and nonmetropolitan noncore, as developed by the NCHS to assess the level of rurality in residential areas.

### Analysis

We conducted survey-weighted descriptive analyses to explore the socioeconomic demographics of HINTS 6 respondents, their susceptibility to social media misinformation, and their responses to health information on social media. This analysis presented the frequencies (n), weighted estimates of the sample, and weighted percentages (%) of various variables. To account for the complex survey design of HINTS, we incorporated jackknife replication weights to adjust for standard errors. In addition, we used ordinal logistic regression analysis to investigate the relationship between predictor variables and respondents' susceptibility to health misinformation on social media. We tested 4 models that examined the associations between 4 variables pertaining to susceptibility and the sociodemographic predictors. As shown in Table 3, Model 1 assessed the extent of exposure to false or misleading health information on social media. Model 2 explored whether respondents used social media information in their health decision-making process. Model 3 examined whether individuals discussed information from social media with health care providers. Model 4 investigated the difficulty individuals faced in assessing health information on social media. The socioeconomic and demographic variables were included as predictors in these models. Respondents with missing values for any of the items were excluded from our analysis. All statistical analyses were carried out using STATA software (version 17.0; StataCorp LLC).

### Ethical Considerations

HINTS data is collected by the National Cancer Institute and is available to scholars for research purposes. No separate approvals were sought for the use of this secondary data as all the data, survey instruments, and methodology reports are available on the HINTS website. Participation in the survey was voluntary. Participants were offered incentives ranging from US $2 in the initial mailing to US $10 or US $30 (for specific groups) to improve response rates. Additional details about the informed consent process, incentives, and methodology are available on the HINTS website [40].

## Results

### Sample Demographics

Our final analysis included 5041 adults (out of the 5922 survey respondents) who indicated using social media. Table 1 presents frequencies, weighted frequencies, and weighted percentages of the demographic characteristics of the study population. The respondents were 51.87% (n=2910) female, 59.77% (n=2553) non-Hispanic White, 11.3% (n=720) non-Hispanic Black, 17.45% (n=835) Hispanic, 5.94% (n=246) non-Hispanic Asian, and 5.54% (n=164) other non-Hispanic adults. Age distribution revealed that 29.62% (n=919) were between 18 and 34 years old, 27.57% (n=1168) were in the 35-49 age bracket, 26.49% (n=1469) were aged 50-64, 10.69% (n=966) were between 65 and 75 years old, and 5.62% (n=464) were 75 years old or older. In addition, 56% (n=2518) were either married or cohabiting. In terms of education, 34.61% (n=2116) held a bachelor’s or postbaccalaureate degree, while 38.98% (n=1366) had completed “some college.” In addition, 20.28% (n=755) had graduated from high school, and 6.12% (n=249) had education levels below high school. Household income distribution indicated that over 46% (n=1873) reported an annual income above $75,000, 18% (n=776) fell within the $50,000 to $75,000 range, 11.38% (n=577) had incomes between $35,000 and $50,000, 10.87% (n=569) earned between $20,000 and $35,000, while 13.63% (n=688) reported incomes less than $20,000. In addition, 31.88% (n=1962) resided in a large metropolitan area, while 46.34% (n=2103) were from either large fringe areas or medium metros. A smaller percentage, 9.85% (n=352), came from small metros, and 11.93% (n=624) hailed from nonmetro, micropolitan, or noncore regions (Table 1).

**Table 1:** Study population demographics.

		Sample, n	Weighted count	Percentage, %
**Birth gender**				
	Male	1771	98790072.75	48.13
	Female	2910	106475217.3	51.87
**Marital status**				
	Not married	2159	90332590.72	44.04
	Married or cohabitating	2518	114794809.3	55.96
**Age group (years)**				
	18-34	919	64862315.77	29.62
	35-49	1168	60382516.95	27.57
	50-64	1469	58018272.31	26.49
	65-74	966	23416142.89	10.69
	75+	464	12300699.69	5.62
**Education**				
	Less than high school	249	12492492.71	6.12
	High school graduate	755	41366798.43	20.28
	Some college	1366	79521119.41	38.98
	Bachelor’s degree	1372	40856012.93	20.03
	Postbaccalaureate degree	944	29746253.58	14.58
**Race\ethnicity**				
	Non-Hispanic White	2553	119961238.7	59.77
	Non-Hispanic Black	720	22673290.6	11.30
	Hispanic	835	35015805.29	17.45
	Non-Hispanic Asian	246	11931031.66	5.94
	Non-Hispanic other	164	11122270.69	5.54
**Household income**				
	<$20,000	688	26819275.74	13.63
	$20,000 to < $35,000	569	21398873.45	10.87
	$35,000 to < $50,000	577	22396522.45	11.38
	$50,000 to < $75,000	776	35433041.84	18.01
	$75,000 or more	1873	90747802.43	46.11
**Location**				
	Metropolitan: large metro	1962	70436347.45	31.88
	Metropolitan: large fringe metro	1108	54876171.34	24.84
	Metropolitan: medium metro	995	47496064.98	21.50
	Metropolitan: small metro	352	21765167.73	9.85
	Non-Metropolitan: Micropolitan	369	15577808.38	7.05
	Non-Metropolitan: Noncore	255	10792811.39	4.88

### Susceptibility to Health Misinformation in Social Media

About 35.61% (1740/4959) of respondents reported finding a substantial amount of health information on social media to be misleading or false. Meanwhile, 45.77% (2256/4959) felt that only some of this information was misleading, while a smaller portion, 17.09% (855/4959), considered 'a little' information misleading. Surprisingly, only 1.54% (108/4959) believed that none of the health-related information on social media was misleading. In evaluating health information found on social media, a significant majority, 66.56% (3225/4928), somewhat or strongly agreed that they encounter difficulty in assessing health information on social media. However, 15.59% (818/4928) strongly disagreed, while 17.85% (885/4928) somewhat disagreed that they had difficulty in assessing the truthfulness of health information on social media (Table 2).

Among the respondents, 16.94% (811/4939) either somewhat or strongly affirmed their use of health information from social media in their personal health decisions. Contrastingly, 60.84% (3090/4939) strongly disagreed with this practice, while an additional 22.21% (1038/4939) somewhat disagreed. When asked about using health information from social media in discussions with their health care provider, 59.18% (2947/4935) strongly disagreed. 20.71% (977/4935) somewhat disagreed, while 20.11% (1011/4935) somewhat or strongly agreed, indicating that they discuss such information with their providers.

**Table 2:** Susceptibility to health misinformation on social media.

		Sample, n	Weighted count	Percentage, %
**How much of the health information in social media you see is false or misleading?**				
	None	108	3342519.182	1.54
	A little	855	37194084.68	17.09
	Some	2256	99611868.69	45.77
	A lot	1740	77499724.54	35.61
**Difficulty in assessing health information on social media.**				
	Strongly disagree	818	33749756.19	15.59
	Somewhat disagree	885	38662314.23	17.85
	Somewhat or strongly agree	3225	144133135.4	66.56
**Use information from social media to make decisions about health.**				
	Strongly disagree	3090	132073094.1	60.84
	Somewhat disagree	1038	48218662.57	22.21
	Somewhat or strongly agree	811	36,776,697	16.94
**Use information from social media in discussions with health care provider.**				
	Strongly disagree	2947	128438034.5	59.18
	Somewhat disagree	977	44951772.36	20.71
	Somewhat or strongly agree	1011	43650679.74	20.11

### Racial and Demographic Disparities

Table 3 displays the outcomes of 4 ordinal logistic regression models that investigated the relationships between susceptibility to social media health misinformation and various demographic variables, including age group, marital status, race and ethnicity, gender, education, annual household income, and location.

In investigating the prevalence of health misinformation on social media (Model 1), non-Hispanic Black (odds ratio [OR] 0.45, 95% CI 0.33-0.60) and Hispanic (OR 0.54, 95% CI 0.41-0.71) individuals, when compared to non-Hispanic White individuals, exhibited lower odds of encountering a substantial amount of health misinformation on social media. This suggests that these 2 racial groups may be more susceptible to health misinformation on social media channels. Individuals aged 75 years or older (OR 0.63, 95% CI 0.43-0.93), in comparison to those in the 18-34 age group, exhibited reduced odds of encountering a significant amount of health misinformation on social media. Furthermore, US adults with annual household incomes ranging from US $50,000 to US $75,000 (OR 1.74, 95% CI 1.14-2.68), as well as those earning more than $75,000 (OR 1.78, 95% CI 1.20-2.66), demonstrated notably higher odds of encountering a substantial volume of health misinformation through social media channels.

In our examination of the difficulties associated with assessing the accuracy of health information on social media (Model 4), we observed an increase in the odds with age. Particularly, individuals in the age groups of 65-74 (OR 1.66, 95% CI 1.11-2.51) and 75 and above (OR 1.86, 95% CI 1.10-3.14), when compared with the younger 18-34 group, displayed higher odds of finding it challenging to assess the truthfulness of health information obtained from social media channels. Furthermore, Hispanic (OR 1.68, 95% CI 1.48-1.98) and non-Hispanic Asian (OR 1.96, 95% CI 1.21-3.18) individuals, when contrasted with non-Hispanic White individuals, exhibit significantly higher odds of encountering challenges in assessing health information sourced from social media.

**Table 3:** Results from ordinal logistic regression analysis.

			Model 1: How much of the health information in social media you see is false or misleading?				Model 2: Use information from social media to make decisions about health.				Model 3: Use information from social media in discussions with health care provider.					Model 4: Difficulty in assessing health information on social media.		
			OR^a^ (95 % CI)		*P* value		OR (95% CI)		*P* value		OR (95% CI)		*P* value		OR (95% CI)			*P* value
**Birth gender (Ref^b^: Male)**																		
	Female	1.03 (0.82-1.28)		.82		0.95 (0.76-1.19)		.68		1.17 (0.94-1.46)		.15		1.09 (0.89-1.33)			.42	
**Marital status (Ref: Not married)**																		
	Married or cohabitating	0.95 (0.75-1.18)		.62		1.00 (0.80-1.26)		.99		1.03 (0.81-1.32)		.80		0.93 (0.71-1.2)			.56	
**Age group (years; Ref: 18-34)**																		
	35-49	0.91 (0.67-1.23)		.52		0.55 (0.41-0.77)		<.001		0.65 (0.51-0.85)		<.001		0.85 (0.62-1.15)			.29	
	50-64	0.98 (0.70-1.38)		.90		0.67 (0.50-0.91)		.01		0.89 (0.66-1.19)		.42		1.17 (0.87-1.59)			.29	
	65-74	1.06 (0.76-1.46)		.74		0.61 (0.44-0.85)		<.001		0.70 (0.55-0.89)		.01		1.66 (1.11-2.51)			.02	
	75+	0.63 (0.43-0.93)		.02		0.57 (0.40-0.81)		<.001		0.67 (0.44-1.03)		.07		1.86 (1.10-3.14)			.02	
**Education (Ref: less than high school)**																		
	High school graduate	0.88 (0.53-1.46)		.61		0.77 (0.41-1.46)		.42		0.68 (0.36-1.31)		.25		1.22 (0.61-2.46)			.58	
	Some college	1.13 (0.68-1.87)		.63		0.90 (0.47-1.75)		.76		0.92 (0.5-1.7)		.79		1.26 (0.67-2.37)			.46	
	Bachelor’s degree	0.86 (0.53-1.41)		.55		0.89 (0.48-1.63)		.69		1.01 (0.55-1.85)		.99		0.93 (0.47-1.85)			.83	
	Postbaccalaureate degree	1.18 (0.69-2.01)		.54		0.79 (0.43-1.44)		.43		1.02 (0.54-1.92)		.96		0.60 (0.31-1.19)			.14	
**Race\ethnicity (Ref: non-Hispanic White)**																		
	Non-Hispanic Black	0.45 (0.33-0.6)		<.001		1.21 (0.90-1.62)		.20		1.01 (0.72-1.41)		.98		0.86 (0.65-1.14)			.29	
	Hispanic	0.54 (0.41-0.71)		<.001		1.64 (1.13-2.39)		.01		1.25 (0.97-1.62)		.08		1.68 (1.48-1.98)			.04	
	Non-Hispanic Asian	0.91 (0.55-1.5)		.71		2.19 (1.18-4.1)		.01		2.09 (1.15-3.82)		.02		1.96 (1.21-3.18)			.01	
	Non-Hispanic other	1.10 (0.54-2.26)		.79		0.51 (0.28-0.93)		.03		0.79 (0.45-1.4)		.42		0.68 (0.39-1.2)			.18	
**HHInc^c^ (US $; Ref: <20,000)**																		
	20,000 to <35,000	1.37 (0.86-2.17)		.18		0.65 (0.41-1.03)		.07		0.55 (0.34-0.89)		.02		0.95 (0.56-1.62)			.85	
	35,000 to <50,000	1.18 (0.80-1.75)		.39		0.54 (0.36-0.84)		.01		0.51 (0.32-0.79)		<.001		0.91 (0.57-1.45)			.68	
	50,000 to <75,000	1.74 (1.14-2.68)		.01		0.60 (0.36-1.02)		.06		0.57 (0.32-1.04)		.07		0.89 (0.59-1.36)			.60	
	75,000 or more	1.78 (1.20-2.66)		.01		0.62 (0.40-0.99)		.04		0.54 (0.32-0.89)		.02		1.18 (0.79-1.75)			.42	
**Location (Ref: Large metro)**																		
	Large fringe metro	1.13 (0.86-1.49)		.39		1.05 (0.75-1.45)		.78		1.01 (0.72-1.43)		.94		1.08 (0.79-1.48)			.60	
	Medium metro	1.34 (0.99-1.81)		.06		1.11 (0.80-1.52)		.53		1.26 (0.93-1.69)		.13		1.02 (0.78-1.33)			.90	
	Small metro	1.34 (0.97-1.84)		.08		0.78 (0.46-1.34)		.37		0.91 (0.54-1.54)		.73		1.01 (0.69-1.48)			.97	
	Micropolitan	1.27 (0.87-1.85)		.21		1.11 (0.73-1.69)		.62		1.16 (0.74-1.81)		.50		1.37 (0.78-2.41)			.27	
	Noncore	0.94 (0.53-1.67)		.82		0.73 (0.42-1.27)		.26		0.95 (0.6 -1.52)		.83		0.83 (0.45-1.55)			.55	

^a^OR: odds ratio.

^b^Ref: reference group.

^c^HHInc: annual household income.

In the analysis of health-related decision-making based on information from social media (Model 2), individuals within the age groups of 35-49 (OR 0.55, 95% CI 0.41-0.77), 50-64 (OR 0.67, 95% CI 0.50-0.91), 65-74 (OR 0.61, 95% CI 0.44-0.85), and 75 years and older (OR 0.57, 95% CI 0.40-0.81), in contrast to those in the 18- to 34-year age group, exhibited significantly diminished odds of basing health decisions on information derived from social media. This suggests that younger adults in the 18-34 age group are more likely to integrate social media–based health information into their decision-making processes regarding personal health matters. Our analysis also revealed significant associations between race/ethnicity and health decision-making based on information from social media. When compared with non-Hispanic White individuals, Hispanic (OR 1.64, 95% CI 1.13-2.39) and non-Hispanic Asian (OR 2.19, 95% CI 1.18-4.10) individuals displayed significantly elevated odds of making health-related decisions based on information obtained from social media. Conversely, non-Hispanic individuals categorized as “others” (OR 0.51, 95% CI 0.28-0.93) exhibited lower odds in this regard. The likelihood of making health decisions based on information observed on social media exhibits a diminishing trend with higher income levels. As indicated in Table 3, the odds decrease as annual household income levels increase.

In regard to incorporating social media–based health information in discussions with health care providers (Model 3), our analysis reveals that non-Hispanic Asian (OR 2.09, 95% CI 1.15-3.82) and Hispanic (OR 1.25, 95% CI 0.97-1.62; *P*=.08) individuals exhibit higher propensities compared to non-Hispanic White individuals. In addition, the odds appear to decrease with both age and income levels. Gender, marital status, location, and educational levels did not emerge as significant in any of the 4 models that we examined.

## Discussion

The pervasiveness of misleading or false health information on social media poses a significant threat to public health. In our study, a good proportion of respondents (35.61%, 1740/4959) reported encountering a lot of misleading or false health information on social media, underscoring the widespread presence of health misinformation on these platforms. We also found considerable racial disparities when examining susceptibility to health misinformation on social media. Interestingly, non-Hispanic Black and Hispanic individuals reported lower odds of finding deceptive information on social media. Previous studies have documented considerable circulation of health misinformation and susceptibility of Black communities [41,42], primarily due to the lack of access to health resources, low levels of health literacy, and barriers due to social determinants of health [43,44]. Susceptibility to health misinformation on social media for Black individuals may not vary much as compared with that of non-Hispanic White individuals, as we did not detect any significant differences in 3 of the 4 models tested. However, our findings also indicated that Hispanic and non-Hispanic Asian groups find it difficult to assess the truthfulness of health information in social media channels. In addition, Hispanic and Asian individuals are more likely to discuss social media–based health information with their provider and also make health-related decisions based on what they find in social media. Previous studies have found Hispanic individuals to face a heightened risk of adverse health outcomes, including obesity, diabetes, and HIV [45]. Previous research has recorded the vulnerability of Asian American individuals to health-related misinformation and its impact on their decision-making regarding health. During the COVID-19 pandemic, misinformation about the pandemic was disseminated among Asian American individuals, including Chinese, Korean, Vietnamese, and South Asian American individuals, and this influenced their vaccine decision-making [46]. Exposure to health misinformation, which suggests that smoking or alcohol consumption can protect against COVID-19, was associated with self-reported increases in tobacco and alcohol use among Chinese individuals [47]. Hispanic communities have also been found to be susceptible to misinformation regarding the safety of COVID-19 vaccines and the efficacy of “alternate treatments” [48]. Further, considerable differences exist in how Hispanic individuals and non-Hispanic White individuals use social media platforms [49]. Taken together, our findings indicate considerable vulnerabilities among Hispanic and non-Hispanic Asian groups, highlighting the need for targeted interventions to address these disparities. As racial groups are more prone to making health-related decisions based on information found on social media, health care providers should specifically engage with individuals from these communities to educate them on health misinformation on social media platforms.

Our findings indicate that individuals of different age groups exhibit varying levels of susceptibility to health misinformation on social media. Older adults aged 65 years and older often face challenges in assessing the credibility of health information encountered on social media. This can be attributed to several factors, including limited exposure to digital literacy education, unfamiliarity with social media platforms, and cognitive changes associated with aging. Furthermore, older adults are less likely to discuss social media–based health information with their health care providers. This may be due to concerns about appearing uninformed or a reluctance to trust information on social media. Consequently, health care providers may miss opportunities to address potential health concerns stemming from misinformation exposure. Our findings also note that older adults, as compared with younger ones, are less likely to make health-related decisions based on information from social media. On the other hand, younger adults aged between 18 and 34 seem more vulnerable and susceptible. The younger adults are likely to spend more time on social media channels and, hence, could get more exposure to misleading health information that could even factor into their health decision-making processes. Studies have shown that younger adults tend to miss physician visits [50], engage in fewer discussions and interactions with their health care providers [51], and are more highly influenced by external sources [52]. This combination of factors makes them particularly vulnerable to the influence of health misinformation on social media. If younger adults continue to rely heavily on social media for health information and fail to seek guidance from health care professionals, they may make uninformed decisions that could jeopardize their health and well-being. This highlights the urgent need for interventions that address the issue of health misinformation on social media and promote informed decision-making among younger adults.

Our study identified a notable connection between income levels and susceptibility to health misinformation on social media. Individuals with higher annual household incomes are prone to encountering a greater amount of misleading information on social media. Paradoxically, despite their exposure, they are less inclined to engage in discussions with health care providers or base critical health decisions on information from social media. Conversely, individuals with lower incomes may be more susceptible to health misinformation, highlighting potential disparities in information access and decision-making resources. Our findings regarding positive associations between income levels and misinformation susceptibility are similar to a few studies [53,54] but differ from others who have recorded a negative association [55,56]. Some researchers have also suggested a curvilinear relationship between income levels and misinformation susceptibility [57]. Individuals with higher incomes may be more susceptible to medical misinformation on social media due to their greater access to technology and trust in authoritative sources without critical evaluation. Busy lifestyles and reliance on social networks could limit their ability to thoroughly assess information, while targeted marketing may exploit their purchasing power. Overconfidence in personal knowledge and peer influence further exacerbate susceptibility, highlighting the need for interventions promoting critical thinking and digital literacy across socioeconomic groups.

### Limitations

This study has some important limitations. First, our focus was on racial and sociodemographic disparities that are likely to be associated with susceptibility to health misinformation. There could be several other factors, like health conditions, health literacy, and technological know-how, which could influence an individual’s behavior toward social media–based health information. Second, data obtained from HINTS rely on self-reported responses that could be biased. Third, the cross-sectional nature of data collection relies on data at one specific point in time. Given the evolving nature of the social media landscape, individual behavior on social media could change with time, and additional data collection across different time periods could throw light on the longitudinal behavioral dynamics. Finally, we were also limited by the use of a predefined set of measures included in the HINTS survey. A self-developed set of measures that is based on theoretical frameworks from cognitive psychology could capture the multidimensional nature of susceptibility to misinformation.

### Implications and Recommendations

Our research highlights the intricate interplay of sociodemographic and racial/ethnic factors in shaping individuals’ interactions with health information on social media. To address the multifaceted challenges identified, targeted health literacy campaigns should be developed, particularly focusing on younger age groups, Hispanic individuals, and non-Hispanic Asian individuals. These campaigns should emphasize the cultivation of critical evaluation skills for assessing the authenticity of health information disseminated through social media platforms. In addition, there is a pressing need to acknowledge and address racial and ethnic disparities by tailoring health communication strategies to different demographic groups. This tailored approach can enhance the relevance and effectiveness of public health interventions.

Furthermore, our study emphasizes the importance of equipping health care providers with the necessary skills and knowledge to navigate discussions about social media–based health information with patients [58]. This not only fosters a collaborative and informed health care decision-making process but also positions health care providers as key advocates in increasing awareness about health misinformation on social media. They can provide specific guidance, especially to vulnerable groups, to navigate the influx of health information online. Health care providers should acknowledge that patients frequently seek medical guidance from the Internet, friends, peers, and family members. Discouraging patients entirely from using alternative sources is unlikely to succeed. To combat misinformation, providers should empower patients by providing accurate information that meets their needs for self-education [59,60].

Another tactic to handle health misinformation dissemination that has emerged has been proactive efforts by social media platforms to label inaccurate content or to lower the prominence of such content in their presentation algorithms. Many individuals have reported encountering interventions such as credibility labels that are assigned to social media posts [61]. Accuracy nudges involving subtle clues or prompts and post hoc corrections of misinformation have helped improve the discerning behavior of individuals toward health misinformation on social media [62,63]. However, evidence regarding whether such efforts affect misinformation spread and acceptance is mixed. Exposure to fact-checking labels has been found to discourage misinformation sharing [64] as well as encourage positive health decision-making behaviors [65], whereas evidence for effects such as reduced acceptance of inaccurate claims is also contested [66]. While the impact of fact-checking labels may be uncertain, community engagement approaches centered on building relationships over time show potential in establishing trusted communication networks. These networks can play a crucial role in combating the spread of health misinformation among specific racial groups and communities. For instance, approaches involving the use of social media influencers, volunteers, and celebrities to spread pro-vaccine messaging on social media platforms have proved to be effective in Hispanic communities [67]. Educational campaigns to combat health misinformation on social media have also been effective [68].

Finally, health care institutions and agencies have a crucial role to play in combating health misinformation. They can contribute by offering fact-check mechanisms, partnering with social media companies to contain the spread of misinformation, and implementing strategies to enhance the digital health literacy of the broader population. These collaborative efforts are vital in creating a robust defense against the unchecked dissemination of misleading health information on social media platforms.

### Theoretical Implications

The theoretical implications of this study are significant, particularly in the context of information processing theories that deal with superficial processing and selective processing. The findings underscore the prevalence of health misinformation on social media platforms, implicating individuals’ susceptibility to misleading information. Specifically, the observed racial disparities suggest variations in how different demographic groups engage with and evaluate health information on social media. For instance, non-Hispanic Black and non-Hispanic Asian individuals may demonstrate a tendency towards selective processing, wherein they are less likely to encounter deceptive information but may face challenges in assessing the truthfulness of health content on social media. Conversely, Hispanic and non-Hispanic Asian individuals may exhibit tendencies toward superficial processing, as evidenced by their increased likelihood to discuss health information with providers and make health decisions based on social media content, potentially without thorough scrutiny.

Furthermore, the age-related patterns observed in the study align with theories of information processing, with older adults exhibiting challenges in assessing health information on social media, possibly due to cognitive factors or limited digital literacy. Conversely, younger adults demonstrate increased vulnerability to health misinformation, suggesting a propensity toward superficial processing or susceptibility to persuasive content on social media. In addition, the linkage between income levels and higher exposure to health misinformation suggests complexities in decision-making and information access, potentially influenced by factors such as digital literacy, access to reputable sources, and trust in information on social media.

### Conclusion

In conclusion, addressing health misinformation on social media requires a nuanced and comprehensive approach that considers the diverse demographic factors influencing individuals’ interactions with online health content. By understanding these complexities, public health initiatives can better tailor strategies to foster informed decision-making and mitigate the impact of misinformation on individual and community health.

## Abbreviations

ACS: American Community Survey
HINTS: Health Information National Trends Survey
NCHS: National Center for Health Statistics
OR: odds ratio
RUCA: Rural-Urban Commuting Area

## Data Availability

Data used in this study is available on the Health Information National Trends Survey website.
